# The risk of digital defencelessness arising from generative artificial intelligence

**DOI:** 10.3389/fpsyg.2026.1807330

**Published:** 2026-04-24

**Authors:** Iván Martín y Ladera, María Pilar Martínez-Ruiz, Natividad Araque-Hontangas

**Affiliations:** 1 Facultad de Ciencias Sociales y Tecnologías de la Información de Talavera de la Reina, Departamento de Economía Española e Internacional, Econometría e Historia e Instituciones Económicas, Universidad de Castilla-La Mancha, Talavera de la Reina, Spain; 2 Departamento de Administración de Empresas, Facultad de Ciencias Económicas y Empresariales, Universidad de Castilla-La Mancha, Albacete, Spain; 3 Facultad de Ciencias Sociales, Departamento de Economía Española e Internacional, Econometría e Historia e Instituciones Económicas, Universidad de Castilla-La Mancha, Cuenca, Spain

**Keywords:** digital privacy, generative artificial intelligence (GAI), legal framework, neuro-rights, structural defencelessness

## 1 Introduction

The emergence of generative artificial intelligence (GAI) has rapidly transformed human interaction in the digital environment. In just a few years, platforms such as ChatGPT, Grok, Gemini, Replika, Pi and Claude have evolved from being technological novelties to becoming part of the everyday lives of individuals across all social strata and educational levels. Overnight, thousands of users around the world have turned to these platforms not only to solve practical tasks, but also to share intimate thoughts, emotions and personal dilemmas (Brooks, 2021; Shank et al., 2019).

This new phenomenon has rendered AI systems a type of “digital confidant,” capable of simulating active listening and creating a climate of closeness. Unlike doctors, therapists or lawyers, however, these interactions lack a protective legal, ethical or professional framework (Etzioni and Etzioni, 2017). Thus, what the user perceives as a safe space for venting is actually an environment of structural vulnerability (Barroso Camiade and Pérez Castrejón, 2025; Carrillo, 2020).

The widespread implementation of AI in people's lives through applications or directly at the heart of the operating systems of mobile devices, personal computers, cars and other consumer devices warrants close study, as confirmed by recent systematic reviews of AI's broad social impact (Villagomez Palacios, 2025), given the lack of regulation, which, added to the massive and transnational nature of AI, opens the door to a new form of digital defencelessness (Roumate, 2024).

It is important to distinguish digital defencelessness from related concepts. Algorithmic dependence refers to the growing reliance on AI systems that may erode cognitive autonomy (Ahmed, 2026). The concept Power asymmetry describes the structural imbalance between corporations that accumulate data and the users who generate it (Crawford, 2021; Zuboff, 2019). Digital vulnerability for its part captures consumers' universal susceptibility to exploitation within digital marketplaces (Helberger et al., 2022). Meanwhile, digital defencelessness, as proposed here, extends these notions: it denotes the specific condition in which users confide intimate information to GAI systems that simulate empathy yet lack conscience, legal accountability or professional duty of care, in a context where existing frameworks fail to provide adequate protection.

To understand this phenomenon, a critical analysis is performed, based on three main axes: digital empathy as a mirage, the erosion of anonymity, and legal loopholes and

parallels with social networks. Finally, we underscore the urgency of articulating a holistic theoretical and legal framework capable of anticipating the “black swan” of algorithmic exclusion (De Almeida et al., 2021; Sundblad and Salaya Kalierof, 2025).

## 2 Digital empathy as a mirage

A key factor explaining the rapid adoption of GAI is its ability to simulate closeness: these models are designed to mimic human conversation patterns, adopting a warm and empathetic tone that progressively adjusts to the user's communication style. The interaction is particularly appealing in contexts of emotional vulnerability, as it conveys the feeling of being with someone who is always willing to listen (Weisker, 2025).

However, this apparent empathy is an algorithmic mirage. GAI systems have no conscience, moral responsibility or capacity for ethical judgement. Their responses are based on probabilistic word predictions, designed to maintain the flow of conversation and maximize interaction. The user projects subjectivity and trust onto an agent lacking the guarantees of a human professional (Chaturvedi, 2025; Srinivasan and González, 2022).

This dynamic has significant psychological implications: the illusion of confidentiality encourages individuals to uninhibitedly open up, sharing intimate confessions that would remain hidden in a real social context. However, the relationship is sustained by a system that cannot guarantee authentic emotional support or adequately safeguard the information received, sometimes with serious security breaches in conversations that users think are private (Croes et al., 2024; Gordon and Turnbul, 2024).

What a decade ago might have been considered a black swan is now a reality, revealing an uncertain future marked by threats to citizens. AI continues to grow and occupy spaces that seemed unthinkable just a few years ago. Its benefits are undeniable, but its social and intellectual risks are equally significant (Kreissl and von Laufenberg, 2024).

The mass adoption of GAI, which is replacing the outdated and more limited virtual assistants of various platforms, is transforming it into an assistant, advisor and even a digital “confidant.” Users' trust in it is almost absolute, believing it possesses more information than a person could accumulate over an entire lifetime (Croes et al., 2024).

This constant interaction, designed to keep the conversation going in all circumstances, produces a massive accumulation of data on habits, from the most trivial to the most intimate. However, this collection lacks a solid, transnational legal guarantee, relying instead on opaque algorithms and terms of use that are seldom read or fully understood (Mirishli, 2025).

For those who underestimate the intrusion into their privacy, consider what occurs every time virtual reality glasses, an immersive headset or a mobile phone camera scan the user's environment. This process gives AI access to everything our eyes see, while presenting itself as a fascinating advance that describes the furniture in the bedroom. In reality, this capability conceals the collection of data on a scale unprecedented in human history (Giaretta, 2024; Wassom, 2014).

Data collection, combined with the incredible processing capabilities being developed in data centers across the globe, serves to catalog, classify and categorize the user. The resulting possibilities are both extraordinary and worrying, especially when these databases are sold, transferred or combined with others, and end up in the hands of AI systems that could influence sensitive decisions, such as access to housing or certain services, based on a person's digital footprint, or on what they have done, decided or posted on social media moments before (Panda, 2025; Sabol, 2025).

## 3 The erosion of anonymity

The user's subjective experience is often accompanied by a perception of anonymity with an interlocutor exclusively available 24 hours a day; what is expressed in front of the screen is understood as intimate and ephemeral. However, every interaction is recorded, stored and potentially cross-referenced with other data sets, creating extremely detailed profiles (Distler et al., 2020; Matulin and Mrvelj, 2018; Photiadis and Papa, 2023).

The scale of the databases is overwhelming. ChatGPT alone processes more than 2.5 billion daily requests (Besora, 2025). These digital footprints allow us to infer behavior patterns, political leanings, emotional vulnerabilities and even health risks. Anonymity is diluted in an ocean of information that, far from protecting the individual, exposes them to invisible mechanisms of social classification (Barocas and Selbst, 2016).

This scenario clashes with the framework of neuro-rights, which seeks to enshrine fundamental rights such as mental privacy (Adán Ríos, 2022), cognitive freedom, psychological integrity and continuity of identity (Ienca and Andorno, 2017; Yuste et al., 2017). The trust and confidence placed in GAI may become a criterion for structural exclusion, effectively undermining these rights. A misinterpreted ironic question could be classified as an indicator of instability; an intimate confession, as a marker of risk.

The result is the emergence of a fertile ground for algorithmic discrimination, which can affect access to employment, credit and basic services (Wachter et al., 2017).

## 4 Legal loopholes and parallels with social media

The current regulatory framework is insufficient to address the challenges posed by GAI. Each query is converted into data that is stored and transferred to different national and transnational jurisdictions, sometimes with lax legislation, hindering any attempt at protection or complaint by users (Altman, 2025). This situation takes place across interconnected legal levels, revealing a deeper structural problem.

In the case of Spain, Organic Law 3/2018 (LOPDGDD; Agencia Estatal Boletín Oficial del Estado, 2018)—which adapts the GDPR (European Union, 2016) to the national legal order and recognizes digital rights in its Title X—highlights these limitations. Specifically, the right to erasure (arts. 93–94) cannot guarantee the deletion of cognitive profiles already embedded in model parameters, and its consent standard (art. 6) was not designed for the continuous, inferential data generation that characterizes conversational AI. In addition, neither the LOPDGDD nor the broader European framework—including the Artificial Intelligence Act (European Union, 2024), in force since August 2024—classifies conversational GAI platforms as high-risk systems. Under Article 52 of the AI Act, they fall within the “limited risk” category, subject only to transparency obligations that operate primarily between providers and downstream developers, without generating directly enforceable rights for the citizen user.

The inherently transnational nature of GAI platforms further exacerbates this regulatory gap. A key factor must be taken into account: the major conversational AI systems are developed and operated predominantly in the United States of America, a jurisdiction which, as of early 2026, lacks any comprehensive federal AI legislation governing user protection. As a result, regulatory efforts remain fragmented across individual states—with California, Colorado, and New York among the most active—without a unified federal standard addressing the psychological or relational risks of conversational AI. In this context, Executive Order 14179, signed by President Trump on 23 January 2025 (*Removing Barriers to American Leadership in Artificial Intelligence*), is particularly significant, as it revoked the previous administration's AI safety directives and explicitly prioritized innovation and economic competitiveness, further reducing the likelihood of establishing federal user-protection standards in this domain in the short term (The White House, 2025). Because of this, users in Europe may interact with systems nominally governed by the AI Act, while their operators remain subject to no equivalent obligations in their home jurisdiction.

International organizations such as UNESCO have adopted the Recommendation on the Ethics of Artificial Intelligence (UNESCO, 2021), applicable to 193 member states, and the more recent (UNESCO 2025) Recommendation on the Ethics of Neurotechnology (November 2025) both of which establish that human dignity and mental integrity must be protected throughout the AI lifecycle, and introduce the concept of neural data as a special category of sensitive data requiring enhanced safeguards. Both instruments remain non-binding. Neither the GDPR, the AI Act, nor the LOPDGDD currently classifies cognitive or emotional data inferred from GAI conversational interactions as equivalent to health or biometric data—a gap that directly undermines the neuro-rights framework proposed by (Ienca and Andorno 2017) and (Yuste et al. 2017), including the specific articulation of the right to mental privacy (Ienca, 2021).

This problem is not new; rather, it replicates dynamics observed in various social networks, where recommendation algorithms have daily reconfigured the public sphere, leading to polarization and the generation of so-called emotional dependency (Pariser, 2011; Zuboff, 2019). The most recent studies suggest that continuous exposure to digital stimuli of immediate gratification directly impacts the brain's reward circuits, while reducing critical capacity (Cavanaugh et al., 2016; Korte, 2020).

GAI amplifies these dynamics. Its conversational systems not only record words, but also use them to reinforce patterns of dependency and shape users' perceptions of themselves. The absence of regulation exacerbates a scenario in which the intimate becomes data, and the confidential becomes merchandise, sometimes sold to the highest bidder (Bravo Galindo, 2025).

## 5 Discussion

Drawing on what (Taleb 2007) calls a predictable black swan, it is possible to foresee that the current scenario resulting from the growing use of GAI may lead to such a phenomenon. In other words, it represents a high-impact outcome whose occurrence is virtually inevitable unless urgent action is taken. Structural digital defencelessness threatens to transform trivial queries into permanent labels, with disproportionate consequences for individuals' lives.

In response, a holistic theoretical and legal framework is needed that goes beyond fragmented technical solutions. Such a framework should integrate legal, ethical, social and philosophical dimensions, in line with the anticipatory governance approach proposed for emerging technologies (Guston, 2014). Priority measures include:

• Protection equivalent to professional secrecy for interactions with GAI.

• The right to be forgotten and to digital contradiction, ensuring that a one-off query does not become a permanent stigma.

• External audits of algorithms, designed to detect bias and unfair exclusions.

• Transparency and informed consent regarding the destination of the data generated.

It is essential, both now and in the future, to close this normative gap. To do so will require binding cross-jurisdictional coordination, not merely voluntary principles. The Council of Europe's Framework Convention on Artificial Intelligence (2024) points toward this goal, but its impact will remain limited as long as the jurisdictions that develop and operate the major GAI platforms continue to resist enforceable international obligations (Council of Europe, 2024).

Beyond regulation, this debate requires philosophical reflection on subjectivity, consciousness and dignity in an environment mediated by machines that simulate understanding. The challenge is 2 fold: to protect individuals from legal defencelessness and to preserve the human condition in the face of technologies that dilute privacy through big data.

In short, society cannot afford to repeat the mistakes made with social media. This time, not only the public sphere is at stake, but also the mental and emotional privacy of every individual. Consequently, this challenge must be urgently recognized as a first step toward a regulatory framework that anticipates risks and safeguards human dignity in the age of AI.
